# Perspectives on the probiotic potential of lactic acid bacteria from African traditional fermented foods and beverages

**DOI:** 10.3402/fnr.v60.29630

**Published:** 2016-03-08

**Authors:** Mduduzi Paul Mokoena, Taurai Mutanda, Ademola O. Olaniran

**Affiliations:** Discipline of Microbiology, School of Life Sciences, College of Agriculture, Engineering and Science, University of KwaZulu-Natal (Westville Campus), Durban, South Africa

**Keywords:** antimicrobials, probiotics, lactic acid bacteria, fermented foods

## Abstract

Diverse African traditional fermented foods and beverages, produced using different types of fermentation, have been used since antiquity because of their numerous nutritional values. Lactic acid bacteria (LAB) isolated from these products have emerged as a welcome source of antimicrobials and therapeutics, and are accepted as probiotics. Probiotics are defined as live microbial food supplements which beneficially affect the host by improving the intestinal microbial balance. Currently, popular probiotics are derived from fermented milk products. However, with the growing number of consumers with lactose intolerance that are affected by dietary cholesterol from milk products, there is a growing global interest in probiotics from other food sources. The focus of this review is to provide an overview of recent developments on the applications of probiotic LAB globally, and to specifically highlight the suitability of African fermented foods and beverages as a viable source of novel probiotics.

Traditional fermentation is a form of food processing achieved by using microorganisms, especially lactic acid bacteria (LAB), and yeast. Although it is an ancient food preservation technology, it is still part of the cultural norm usually being practiced at a local or household level among indigenous communities in Africa and most of the developing world (1–3). Unlike their unfermented counterparts, fermented foods are preferred by consumers because of their characteristic taste, texture, and color (4). Fermented traditional foods and beverages in developing countries constitute one of the main dietary components, with some being used as light meals or refreshments.

LAB isolated from various fermented foods produce organic acids and a high diversity of antimicrobial agents, which are responsible for the upkeep of quality and the palatability of fermented foods. At a local level, LAB are used in African communities to produce a variety of fermented foods which include cereal-based fermented porridges, beverages, fermented fruits and vegetables (including roots or tubers), fermented milks, and fermented meats (4, 5). Of the various types of fermentations used to obtain fermented foods and beverages, lactic acid and alcoholic fermentations are the most popular. Other types are alkaline fermentation and amino acid fermentation (5).

The role of LAB in improving the shelf life and nutritional quality of fermented foods and beverages, controlling diarrhea, as well as their antimicrobial properties have been established (6–9). However, despite an increasing interest in probiotic LAB, there is a paucity of literature regarding novel and emerging uses of LAB as probiotics, especially from the African continent. In addition, strict regulation by international bodies has resulted in limited probiotic products passing the clinical trial stage. Rural communities are known to be consuming fermented foods laden with probiotic microflora, from which they derive health benefits. This paper reviews different topical traditional fermented foods and beverages in Africa which are potential sources of novel probiotics, and explores global progress on the isolation of probiotic LAB candidates. The applications of LAB as antidiabetic agents, cholesterol-reducing agents, immune system modulation agents, and as drug-delivery vehicles, as well as their novel applications in mental and emotional well-being of humans were discussed in detail.

## Food and beverage fermentation by LAB

Fermented foods have been part of the human diet since the dawn of human civilization, and have been used as a means of improving the shelf life, safety, digestibility, and nutritional value for more than 6,000 years (10–16). Lactic acid food fermentation is a process whereby microorganisms and their enzymes are used to convert fermentable sugars in the food substrate into mainly lactic acid and other limited products. Fermentation is widely used for food preservation in households and in food industries to develop a variety of food products (13). This food preservation technology is inexpensive; hence, it is of economic importance in developing countries (4). Microorganisms responsible for the fermentation can be the microflora indigenously present on substrates, or they can be added as starter cultures after cooking or preparing the food or beverage.

Fermentation of traditional foods is mainly carried out by LAB, which has earned the ‘generally regarded as safe’ (GRAS) status. Fermentation improves the organoleptic properties of foods and their acceptability. Popular fermented food products in Africa include fermented milks, sour porridges, and alcoholic and non-alcoholic beverages (17). Cereals are a staple diet globally and they can be processed through fermentation to acquire desirable modifications of taste, flavor, acidity, and food digestibility (4). There is a vast diversity of African traditional fermented foods and beverages distributed throughout the continent, with typical examples presented in Table 1.

**Table 1:** Various African traditional fermented foods and beverages

Fermented product	Region	Raw substrates	Microorganisms implicated	Published references
*Ogi*	West Africa	Maize, sorghum, millet	*Lactobacillus plantarum* *L. fermentum*, *S. cerevisiae* *Candida krusei*, *Corynebacterium* spp., *Acetobacter* spp.	(23, 117)
				
*Iru*	West Africa	African locust bean	*Bacillus*, *Staphylococcus* spp.	
*Gari*	West Africa	Cassava	*Leuconostoc mesenteroide* *Lactobacillus plantarum* *Bacillus subtilis*, *Candida krusei*	
*Mahewu*	Southern Africa	Maize or sorghum	*Lactobacillus bulgaricus*, *L. brevis*	(7, 9, 17)
*Munkoyo* (*chibwantu)*	Southern Africa	Maize	*Weisella* and *Lactobacillus* spp.	(24, 25, 27)
*Borde* *Shamita*	North East Africa	Barley	*Lactobacillus* spp.	(118)
*Togwa*	East Africa	Cassava, maize, sorghum, millet	*Lactobacillus* spp., *Pediococcus pentosaceus*, *Weissella confusa*, *Issatchenkia orientalis*, *S. cerevisiae*, *Candida pelliculosa* and *C. tropicalis*	(25)
*Mabisi/Amasi*	Southern Africa	Milk	*Lactococcus, Lactobacillus* and *Streptococcus* spp., *Leuconostoc* spp.	(17, 119)
*Amabere amaruranu*	Southern Africa	Milk	*Streptococcus thermophilus*, *Lactobacillus plantarum*, and *Leuconostoc mesenteroides; Yeasts*	(120)
*Marula/buganu*	Southern Africa	Amarula fruit	Yeasts	(9)
*Bushera*	East Africa	Sorghum or millet	*Lactobacillus* spp., *Streptococcus* spp., *Leuconostoc* spp., *Pediococcus* spp., *Weissella* spp.	(121)
*Chibuku*	Southern Africa	Sorghum	*Lactobacillus* spp., *Saccharomyces cerevisiae*	(9)
*Umqombothi*	Southern Africa	Maize or sorghum	*Lactobacillus* spp., *S. cerevisiae*	(122)

In addition to the various fermented plant products, fermented meats and fish form part of the African diet and also of diet in other parts of the world (18–21), although these are arguably not yet common. Nonetheless, fermented meats can be adequate purveyors of probiotic bacteria if properly applied. Fermented meat products are defined as meat that is inoculated with a microbial starter culture during processing under controlled conditions to give desirable characteristics. Meat fermentation can also be achieved by allowing it to spontaneously ferment by natural meat microbial flora. A recent review (22) discusses the application of novel technologies and microbial cultures in the development of functional fermented meats. Zakpaa et al. (19) established that the microbial composition of Ghanaian fermented meats consists of LAB species, *Streptococcus* spp., *Staphylococcus* spp., and *Micrococcus* spp. Of these, *Streptococcus* and *Staphylococcus* spp. are characterized as pathogenic, and the LAB species and *Streptococcus* spp. as probiotics. These results suggest that using a robust probiotic starter culture might be useful in improving the quality of fermented meats, as it may inhibit the growth of potentially pathogenic organisms. Fermentation is one of the most important fish preservation methods in many parts of the world and particularly in the coastal regions of African countries (21). The fermentation processes applied are generally indigenous and adaptable to the culture of the people. However, lack of standardization of the processing methods and hygiene levels compromise the safety and quality of fermented fish products in Africa. Thus, more research needs to be dedicated to finding solutions to these issues.

Fermented foods are produced using different manufacturing techniques, raw materials, and microorganisms depending on available raw materials and local practices. However, the four main fermentation processes are alcoholic, lactic acid, acetic acid, and alkali fermentation. While a majority of these fermentations are carried out by bacteria, in alcohol fermentation, yeasts are the predominant microorganisms used for production of ethanol-containing beverages, such as wines and beers, where LAB play a limited role (4).

LAB are critical in the production of most fermented foods and beverages, but there is a paucity of knowledge about the specific health benefits such foods confer on consumers and the characteristics of the bacterial strains used for their production. Moreover, literature on probiotic microbes from different fermented foods from Africa that have found applications in the food and pharmaceutical industries is scarce.

### Diversity of African traditional fermented foods and beverages and associated microbes

*Ogi*, *iru*, and *gari* (Table 1) are the Nigerian fermented foods that have been commercialized; the rest are still being produced at the household level. *Ogi* is a fermented product of maize or sorghum or millet grains. *Iru* is a fermented product of African locust bean; and *Gari* is a fermented cassava product, produced by peeling fresh roots, grating them into mash, and putting in sacks for fermentation (23). The main microorganisms used for the fermentation in these products are LAB and yeasts.

*Borde* and *Shamita* (Table 1) are important Ethiopian traditional fermented beverages, produced by an overnight fermentation of certain cereals predominantly by LAB (24). LAB associated with *Borde* and *Shamita* fermentation have antimicrobial properties against various food-borne pathogens; the inhibitory products are extracellular and diffusible. *Togwa* (Table 1) is a Tanzanian fermented beverage that could be prepared from cassava, maize, sorghum, and millet, or their combinations. Yeasts and LAB are the predominant microorganisms found in *togwa* (25).

*Amasi* or sour milk, *umqombothi* or sorghum beer, and *amahewu* (Table 1), a non-alcoholic fermented maize meal, are South African foods and beverages. *Amasi* is produced by using specific LAB such as *Lactobacillus delbrueckii* and *Streptococcus* spp. *Amahewu* is produced with LAB starter culture, whereas *umqombothi* is produced from maize or sorghum via wild yeast fermentation, and LAB from malted sorghum adjuncts play a limited role (7). Fermented beverages such as those obtained from amarula fruits using yeast and LAB are popular in parts of Limpopo and Mpumalanga Provinces in South Africa.

Sour porridge is maize or sorghum, which is fermented using mainly LAB to improve and develop palatability, flavor, and nutrition (26). *Chibuku* (Table 1) is a Zimbabwean traditional sorghum beer commercially produced by yeast fermentation of sorghum (9), whereas *mabisi*, *munkoyo*, and *chibwantu* (Table 1) are Zambian traditional food and beverages produced through yeast and lactic acid fermentations (27).

Production of these traditional fermented foods depends on the availability of infrastructure to store and transport the product. Isolation and screening of microorganisms from natural sources has been the most effective means for obtaining robust and genetically stable strains for industrially important food products (28, 29). However, the microbial diversity of the different fermented foods from different localities in Africa still needs to be characterized using more reliable tools such as next generation sequencing and other molecular techniques. This will enable the selection of more robust, probiotic microorganisms with desirable characteristics to be used as starter cultures and will ensure the safety of the foods and beverages and pharmaceutical products.

### Role and importance of LAB in traditional fermented foods

#### General role of LAB

Fermentation may be the most simple and economical way of improving cereal nutritional value, sensory properties, and functional qualities available at the local community level (15, 30). Lactic acid fermentation of cereals has been used as a strategy to decrease the content of antinutrients, such as phytate and tannins, and for improving the bioavailability of micronutrients (31). Many bacteria associated with fermented foods produce antimicrobial bioactive molecules, such as hydrogen peroxide, organic acids, and bacteriocins, that make them effective biopreservatives (12, 32) and produce nutraceuticals to create functional foods with increased bioavailability of nutrients (33, 34). Table 2 presents an overview of some of the various applications of LAB isolates.

**Table 2:** An overview of some of the applications of selected LAB strains

LAB Strain(s)	Applications	Published References
*Lactobacillus rhamnosus*	Reduction of dental caries risk	(63, 123)
*L. casei*	Mitigation of Type II diabetes and obesity	(67, 124)
*Bifidobacterium longum* SPM 1207; *Lactobacillus* spp.	Lowering of cholesterol and low-density lipoproteins	(77, 80, 83)
*Lactobacillus* spp.	Inhibition of *Listeria monocytogenes* & *Clostridium* spp.	(20, 90)
*Lactobacillus reuteri; L. plantarum*	Antifungal agents	(3, 20, 92)
*L. casei; L. planturum*	Drug-delivery vehicles	(93, 97, 98, 101)
*L. acidophilus*, *L. salivarius*, *L. rhmnosus*, *L. brevis*, *L. casei*	Immune system modulation and mental health	(39, 42, 53, 115)
*Lactobacillus* spp.	Reduction of mycotoxins in fermented maize products	(7)

#### LAB as probiotics

LAB comprise a significant component of the human gut flora and have several beneficial roles in the gastrointestinal tract. Thus, a better understanding of the intestinal microbial populations will contribute to the development of new strategies for the prevention and/or treatment of several diseases (35). Probiotics are live microbial food supplements which beneficially affect the host by improving the intestinal microbial balance (36–38). Probiotic LAB belong to the genera *Lactobacillus*, *Bifidobacterium*, *Enterococcus*, *Lactococcus*, *Streptococcus*, and *Leuconostoc* (39, 40). Probiotics are consumed in the form of yoghurt, fermented milks, or other fermented foods (37, 41). These products containing living microorganisms have been traditionally used to restore gut health (42). Thus, bacteria known as probiotics because of their positive impact on human health and well-being have been added to various foods for the past decades (42, 43).

Probiotic bacteria from the genus *Lactobacillus* are inherently present in fermented foods and many diseases caused by pathogens invading the gastrointestinal tract can be prevented by maintaining proper intestinal flora by consuming probiotics or fermented foods. Probiotics have a great potential for improving nutrition, soothing intestinal disorders, improving the immune system, optimizing gut ecology, and promoting overall health because of their ability to compete with pathogens for adhesion sites, to antagonize pathogens, or to modulate the host's immune response (33, 43, 44).

*Bifidobacterium* species are strictly anaerobic, inhabit the large intestines, and more than 30 species have been identified. These microbes are added in baby formula milk and dairy products such as yoghurt or their growth in the large intestines is stimulated by prebiotics from diet. They assist in the metabolism of lactose, generation of lactate ions from lactic acid, and vitamin synthesis (40, 45). Generally, probiotic bacteria do not colonize the human intestinal tract permanently, but some strains are able to transiently colonize and modulate the indigenous microbiota (42). LAB are highly beneficial organisms for humans and their use should be promoted for good health.

Candidate probiotic microbial strains must satisfy the criteria for safety, production or manufacturing, administration and application, and survival and colonization in the host (46). *In vitro* experiments are used to investigate whether the selected microbial strains fulfill these criteria and enable screening of microorganisms for their potential as probiotic strains (46). Potential probiotics are further subjected to *in vivo* experiments to validate their efficacy. In this regard, it has been experimentally demonstrated that feeding day-old chicks with a commercial LAB-based probiotic culture after they were challenged with *Salmonella enteritidis* or *Salmonella typhimurium* significantly reduced recovery of *Salmonella* (47). Similarly, it has been demonstrated that fish fed with probiotic-supplemented diets presented significantly higher growth rates and feeding performance than those fed with control diet (48).

Probiotic LAB isolated from fermented food samples exhibit excellent antimicrobial activity against pathogenic bacteria and fungi (3). Bacteria with such notable probiotic properties can be used as a potential source of probiotic for use in pharmaceutical preparations, and as functional foods for the betterment of public health. Developing countries in Asia and Africa have a rich diversity of fermented foods and beverages which can be exploited for isolation of such novel probiotics.

#### The role of LAB in immune system modulation and mental health

Most probiotic foods contain LAB belonging to the genera *Lactobacillus* but not exclusively (39). Certain strains of probiotics are able to stimulate and regulate several aspects of the natural and acquired immune response, although differences in the ability of *Bifidobacterium* and *Lactobacillus* strains to influence the functioning of the immune system have been reported (33). The interactions of these probiotics and their unique metabolic capabilities are critical to their ability to colonize the gastrointestinal tract and immune system modulation.

More than 100 species of *Lactobacillus acidophilus*, *Lactobacillus salivarius*, *Lactobacillus rhamnous*, *Lactobacillus brevis*, and *Lactobacillus casei* have been characterized and identified. They are implicated in enhancing the innate and acquired immunity as well as cause inhibition of proinflammatory mediators (49). Probiotic protective microorganisms prevent pathogens from adhering to the intestines by competing for substrates and places of adhesion, simultaneous production of antibacterial molecules, and stimulating the production of specific antibodies and mucus. Thus, early colonization of the gut with probiotic bacteria is critical for the development of the gut protection barrier.

Several studies have indicated that LAB play a positive role in modulating the host immune system and display of antimicrobial activities against common food-borne pathogens and in preventing and treating diarrhea (11, 28, 33, 34, 50, 51). The current understanding of the role of probiotic bacteria in the human and animal systems is preliminary, partly because of the complexity of the gastrointestinal ecosystem and the increasing variety of strains considered as probiotics (52). However, these mechanisms may be multifactorial and each probiotic strain may have specific functions affecting the host. Specific probiotic bacteria are known to modulate local and systemic immune responses, although the mechanisms of immune modulation are still not fully elucidated. Nevertheless, there is an understanding that bacterial components are recognized by the immune system through their interaction with specific receptors, resulting in the modulation of the immune responses (42).

Another study (53) suggests that there is a connection between microbes inhabiting the human GUT and many aspects of physiology, including brain health. The authors point out that fermentation alters dietary compounds in food and maintain that fermentation-enriched chemicals (lactoferrin, bioactive peptides) and the newly formed phytochemicals such as unique flavanoids may act upon the human intestinal microflora. They further suggest that consumption of fermented foods has a potential to positively affect human health and mental well-being by stimulating the actions of the GUT microflora, via the production of bioactive chemicals and neuropeptides that manifest behaviourally through magnified antioxidant and anti-inflamattory activity, and reduction of intestinal permeability. Other studies established that agmatine and other polyamines associated with fermented meats, fish, and certain beverages have benefits related to brain health (54, 55). All these studies highlight the great strides already made on the role of probiotic foods and beverages on human physiology.

### Potential role of LAB as a remedy for lifestyle health problems

#### Prevention and treatment of dental caries

Evidence suggests that species of *Lactobacillus* and *Bifidobacterium* may have beneficial effects in the oral cavity by inhibiting cariogenic bacteria (54, 56, 57). However, scarcity of information on the probiotic action in the oral cavity suggests that more studies should be conducted on the colonization of mouth by probiotics and their potential effect on and within oral biofilms. With increasing antibiotic drug resistance, the concept of probiotic therapy is compelling and merits further investigation in the field of oral medicine (58–60).

The potential application of probiotic bacteria as a remedy for oral infections is of research interest, and clinical studies carried out thus far suggest that probiotics could be critical in preventing and treating dental caries, periodontal disease, and halitosis (43). *Streptococcus mutans* is regarded as the main cause of dental decay and various lactobacilli are associated with progression of the lesion (61, 62). In one study (63), *Lactobacillus rhamnosus* GG, ATTC (LGG) was demonstrated to be antagonistic to many bacteria, including *S. mutans*, which is responsible for tooth decay. This study shows that consuming milk containing *L. rhamnosus* GG significantly reduces the risk of caries. The effect of probiotics on dental caries has been reviewed elsewhere. Although these studies indicate a positive role of probiotics in oral health, more studies need to be conducted to evaluate the efficacy of several probiotics for this purpose.

A survey of novel technologies for the prevention of dental caries has been conducted, and these include the use of probiotics (64). Dental caries and their biofilms can be treated by using probiotic lactobacilli and bifidobacteria from dairy products, tablets, lozenges, and chewing gum in various dose regimens. Results from randomized clinical trials are promising although further large-scale trials with orally derived anticaries bacteria are essential for the success of bacteriotherapy (65). Most reccently, probiotics have proven to be effective in dealing with dental caries (66). However, more studies are needed to explore the use of probiotics appropriately in the field of dentistry.

#### Prevention of Type II diabetes and obesity

Earlier studies suggest that probiotic bacteria play an important role in management of insulin-independent diabetes, with one study particularly indicating that the oral administration of *L. casei* in mice causes a decrease in plasma glucose levels (67). Nutrients with prebiotic and probiotic properties have positive consequences for host well-being with regard to obesity and diabetes. Bacterial metabolism of nutrients in the gut is suggested to influence the release of bioactive compounds which interact with host cellular targets to control energy metabolism and immunity, resulting in less fat mass development, diabetes, and low levels of inflammation associated with obesity (68). A human randomized, double-blind, placebo-controlled trial concluded that probiotic bacteria induce beneficial changes in gut microbiota, reduce the systemic inflammatory state through altering systemic endotoxin levels, thereby reducing the systemic inflammatory response observed in type II diabetes mellitus subjects (69).

Studies with mice indicate that antidiabetic activity of probiotic LAB could emanate from continuous reduction of the blood glucose through the suppression of glucose absorption from the intestine, thus indicating that specific strains of LAB are crucial for the management of type II diabetes (70). Consumption of yoghurt, rich in probiotic bacteria, may exert antidiabetic and antioxidant properties in human subjects and human microflora and is likely to have a major role in maintaining the homeostasis of human metabolism, suggesting that probiotics intake is a useful approach for modulation of human microbiota (71). Elsewhere, it has been established that probiotic soy milk could modulate blood lipoprotein levels and thus may be considered in managing diabetes complications and atherosclerotic risks (72).

#### Lowering serum cholesterol levels

Cholesterol plays a significant role in human heart health. However, because the human body produces enough cholesterol, dietary cholesterol is extraneous and is a leading cause of cardiovascular diseases such as stroke and heart attack (73). Studies conducted to explore the role of probiotic bacteria in the reduction of serum cholesterol showed promising results where cholesterol precipitated out during *in vitro* studies with a possibility of the same being excreted under *in vivo* conditions (74). However, more research needs to be conducted in this regard as these studies were inconclusive. Moreover, some authors warn that overconsumption of prebiotics and probiotic bacteria could cause intestinal discomfort, and the risk of probiotics being purveyors of antibiotic resistance genes needs further investigation (75).

According to one study, there is a difference in fecal cholesterol-associated short chain fatty acids pattern in conventional rats fed with the oat-based diets with probiotic bacteria in comparison to the group fed with rice-based diet (76). Probiotic bacteria able to reduce cholesterol in blood can be obtained from carnivores because they normally eat meat containing high fat and rarely develop cardiovascular conditions. Isolation and characterization of such strains of bacteria has a potential application in controlling cholesterol levels in humans (73).

*Bifidobacterium longum* SPM 1207 is able to reduce serum total cholesterol and low-density lipoproteins which are associated with bad cholesterol levels, while slightly increasing serum high-density lipoproteins, which are associated with good cholesterol. This suggests that a combination of LAB from the genus *Bifidobacterium* and *Lactobacillus* could result in robust probiotics as the latter genera produce bacteriocins (77–79). A *Lactobacillus oris* HMI68 was successfully isolated from a mother's milk and this strain demonstrated cholesterol-reducing property and was regarded as a candidate probiotic (80).

Interestingly, *Lactobacillus plantarum* and *L. brevis* from pickle cabbage decreased levels of serum cholesterol, total glycerides, and low-density lipoprotein cholesterol but increased high-density lipoprotein cholesterol levels in mice treated with the probiotic bacteria (81). The study suggests that in addition to lowering blood cholesterol, the bacterial strains have the potentials to enhance the activities of antioxidant enzymes, relieve lipid-induced oxidative stress, and serve as probiotics. In addition, *Lactobacillus* spp. and *Weissella* spp. isolated from fermented Indian koozh and cucumber exhibited cholesterol-reducing potential (82).

Recently, the effect of probiotic consumption on the Iraqi human body weight and lipid profile was investigated and probiotic strains showed lowering effects on abdominal adiposity, body weight, and other measures of subjects with obese tendencies, suggesting a positive influence on metabolic disorders (83). In particular, this study showed that consumption of probiotic strains reduces total cholesterol and low-density lipoproteins while increasing levels of high-density lipoproteins. This counteracts obesity effects and regulates lipid metabolism.

## LAB as a source of antimicrobial agents

### Bacteriocins from LAB

Bacteriocins are a heterogeneous group of potent antimicrobial peptides primarily active against closely related organisms, mostly gram-positive bacteria (84). They are ribosomally synthesized during the primary phase of growth (85). Bacteriocins are small cationic molecules of about 30–60 amino acids, forming amphiphilic helices and stable at 100 °C for 10 min. Thus, they vary in spectrum of activity, mode of action, molecular weight (MW), genetic origin and biochemical properties (86). They are classified into three groups: lantibiotics, non-lantibiotics of low MW, and non-lantibiotics of higher MW (87). LAB strains that produce bacteriocins protect themselves from their own toxins by the expression of a specific immunity protein which is encoded in the bacteriocin operon (88).

In fermented foods, LAB display various antimicrobial activities, including bacteriocin production. Consequently, several bacteriocins with potential industrial applications have been purified and characterized. Bacteriocin-producing cultures have been applied to inhibit *Listeria monocytogenes* and *Clostridium* in various fermented meats, vacuum-packaged products, and in vegetable-based foods (20, 84). Bacteriocins can be vital when cold chain is interrupted during transportation of heat-sensitive foods. Artificial chemicals are currently widely used to limit food spoilage organisms, but their potential health risks has led researchers to seriously consider the possible use of bacteriocins or their synergistic effect with chemicals, phenolic compounds, and antimicrobial proteins (86, 89).

The spread of antibiotic resistance and demand for food products with fewer chemical preservatives necessitates the search for new alternatives to avoid the abuse of therapeutic antibiotics (87). LAB isolated from home-made fermented vegetables produce antibacterial substances against both gram-positive and gram-negative common food-borne bacterial pathogens. This broad spectrum of inhibition suggests that the LAB strains have a potential to be used as natural biopreservatives in various food products.

Bacteriocins are mainly bactericidal, with some being bacteriostatic, rendering them useful in the food and pharmaceutical sectors. Their site of action is the bacterial cytoplasmic membrane and on energized membrane vesicles to disrupt the proton motive force (84, 86). However, their drawback could lie in the fact that they inhibit mainly closely related organisms, implying that they may inhibit other desired starter cultures, and may not be active against gram-negative pathogens and food spoilers. Bacteriocins have been found to be effective against gram-positive toxigenic and pathogenic bacteria. Addition of chelating agents has been effective in rendering gram-negative bacteria susceptible to bacteriocins (86). A recent study has established that the use of heterofermentative *Lactobacillus* spp. in the production of Dutch-type cheese-like products reduces the populations of technologically harmful microorganisms that negatively affect the quality, safety, and shelf life of the end products (90).

### Antifungal agents and other antimicrobial agents from LAB

As the number of antibiotic-resistant microbial species increases, fungi and yeasts are no exception, and both human fungal pathogens and spoilage molds in food and feeds are becoming resistant. Interestingly, yeasts and molds are becoming resistant also to preservatives such as sorbate and benzoate as well as chemical detergents (91). LAB produce various antimicrobial compounds such as lactic acid, propionic acid, and diacetyl, which cause pH reductions that inhibit many microorganisms, including fungi (91).

In addition to other functions, *Lactobacillus reuteri* produces reuterin, which has a broad spectrum activity against protozoa, fungi, and both gram-positive and gram-negative bacteria and reuterocyclin, which is a negatively charged, hydrophobic, broad spectrum antibiotic which is restricted only to work against gram-positive bacteria (92). Organic acids, bacteriocins, and antifungal peptides produced by microorganisms can also be isolated from grass silage. *L. plantarum* isolated from grass silage exhibit antifungal activity by producing organic acids, other molecular mass metabolites, and cyclic dipeptides (20).

## LAB as drug-delivery vehicles

Because of their properties such as GRAS status, adjuvant profile, mucosal adhesion, and low intrinsic immunogenicity, LAB have been adjudged to have a potential as a carrier for oral immunization, where the adjuvant function of different *Lactobacillus* species was explored. In one such study (93), *L. casei* and *L. plantarum* consistently showed strong adjuvanticity in different experimental model systems. These results thus demonstrated that the generation of *Lactobacillus*-based live vectors as vaccines instead of pathogen-derived live vaccines is feasible (93). This represents preliminary immunological data of recombinant LAB strains expressing antigens from pathogens.

Live bacterial vaccine vectors can be applied for antigen delivery (94). However, increased stability and low production costs should be pursued in addition to the multiple advantages associated with the use of bacteria as vaccine vectors in order to reap maximum benefits. There is growing interest regarding the use of peptides from LAB in medicinal and personal care products, but there is paucity of clinical data to substantiate their efficacy.

Targeted drug delivery to the colon using probiotics, alone or in combination with prebiotics, ensures direct treatment at the disease site leading to lower dosing and fewer systemic side effects (95). As the habitat of probiotics, the colon is better placed as a portal for the entry of drugs into the circulation system (96). While it has been suggested that muco-adhesive polymers such as lectins and fibrin can be used to improve drug delivery through different routes such as gastrointestinal, nasal, ocular, buccal, vaginal, and rectal routes, LAB are more ideal candidates for drug delivery, especially in the gastrointestinal and nasal routes (97). In addition, LAB have potential as an alternative approach to the treatment of human asthma (98).

Nasal vaccination using live bacterial vectors, where attenuated respiratory pathogens are applied, is more effective than non-colonizing microbes such as LAB. However, in contrast to systemically administered vaccines, recombinant live bacteria generally induce strong mucosal immune responses in the respiratory tract and distal mucosal sites. Although many applications of LAB as host factory and delivery vehicles need further research (99, 100), the already established safety profile and efficiency of this system purports a promising future of GRAS bacteria as expression vectors that can revolutionize the field of recombinant protein expression.

The two main advantages of using probiotics as an alternative approach to health care management are their inherent diverse mechanism of action and being living organisms. The current challenges with this system are probiotic strain identification, characterization, screening, and understanding its mechanism of action for a particular disease. Several approaches including microencapsulation and use of compatible material have been studied with a view to circumvent these challenges (101).

## Major factors impacting on the use of LAB as probiotics

Probiotics are living and non-pathogenic bacteria that have beneficial effects on their host's health (33, 102). The major interest in LAB is driven by the fact that upon consumption, these microorganisms are beneficial to the host by boosting the good microflora of the gastrointestinal tract (GIT), and because probiotics are normally associated with LAB, it makes sense to use traditional fermented foods as the main source of probiotics (102, 103). Probiotic bacteria are commonly marketed as fermented foods and dairy products are popular carriers of probiotics (33, 104). Fermented foods are well suited to promote the positive health image of probiotic bacteria because fermented cereals and dairy products already have a positive health image; consumers are familiar with the fact that fermented foods contain microorganisms; and probiotics used as starter cultures combine the positive images of fermentation and probiotic cultures (104).

Probiotic food products are conventionally sold in the form of fermented milk because milk has been accepted as a nutritious and healthy product, and as an important source of calcium, proteins, phosphorus, and riboflavin (39). However, with an increase of more health conscious consumers in developed countries, there is a demand for non-dairy probiotic fermented food products because some consumers are negatively affected by the lactose and cholesterol content associated with fermented dairy products (39, 105–107). The fat content, sustained viability, and numbers of probiotic bacteria are some of the factors currently affecting the use of LAB as probiotics. Several factors that are considered in the criteria for the selection of candidate probiotic LAB are briefly described below.

### Bile and low pH tolerance

LAB with high tolerance for low pH and bile salts demonstrate the ability to survive in the stomach and intestine, and to successfully compete with other bacteria in this environment and to colonize the GIT of the host. The probiotic potential of six LAB strains was evaluated with respect to tolerance for acidity and to bile salts. All six strains were resistant to pH 3.0 and pH 4.0 after 24 h of incubation showing viability rates of 10^9^ CFUml^−1^ and 10^12^ CFUml^−1^, respectively. Moreover, all six strains also demonstrated a high resistance to bile salt concentration of 0.3% (w/v), giving a cell viability of 10^5^–10^8^ CFUml^-1^ after 24 h of treatment (38). High exopolysaccharide-producing LAB such as *Streptococcus thermophilus* strains exhibit a higher tolerance to bile salts than the *Lactobacillus delbrueckii* subsp. *bulgaricus* strains (108).

### Starter culture populations

Probiotics are live microorganisms providing benefits to humans when they are consumed in adequate amounts. To effect these benefits, fermented foods must contain probiotic microorganisms in populations above 10^6^ CFUml^−1^ or g throughout its shelf life. Accordingly, consumption of more than 100 g per day of a typical commercial bio-yoghurt containing more than 10^6^ CFUml^−1^ is necessary to satisfy adequate numbers of viable cells (109). High population of probiotics in commercial fermented milk products is achieved by adding them during or after the fermentation process separately from the starter culture. For traditional fermented foods, this implies that they must be produced by ensuring probiotics populations of not less than 10^6^ CFUml^−1^, otherwise they might not provide maximum benefit to consumerism (110).

### Adhesion to gut epithelial tissue

Candidate probiotic LAB isolates should demonstrate the ability to adhere to gut epithelial tissue and to colonize the GIT. The relative importance of these characteristics is highlighted by the fact that many of the probiotics available, except *L. rhamnosus*, do not colonize their targeted hosts and consequently do not remain in the GIT for any significant period of time (3, 111, 112).

### Antimicrobial activity and safety

A typical probiotic strain should exhibit antimicrobial effects by producing some of the several compounds such as organic acids, fatty acids, hydrogen peroxide, and diacetyl (112). Probiotics should improve safety to the host by inactivating pathogens but must not demonstrate any pathogenic or toxic activities to the host. In addition, candidate probiotics must have no transferable antibiotic resistance genes and must prevent the production of biogenic amines from dietary proteins (3, 113).

### Other selection criteria for probiotic LAB

Other properties an ideal probiotic LAB candidate must display include resistance to antibiotics, antimutagenicity properties, rapid production of lactic acid, viability and retention of activity in delivery vehicles, ability to stimulate the host immune response, and the ability to influence metabolic activities such as vitamin production, cholesterol assimilation, and lactose metabolism (3, 114).

## Current status and future prospects of probiotic bacteria

Probiotic is the current buzz word in the human diet arena and has captured major attention from researchers across the world, including developing countries, because of its enormous health potential (115, 116). The term probiotics was first used in 1965 and defined at that time as microbially derived factors that stimulate growth of other organisms. However, in 1989 the term probiotics was modified to mean live microbes that are beneficial to the host (115). Although probiotic microorganisms have been used for centuries in food production, global research on their products and applications has recently began. There is an increasing interest in utilizing probiotic LAB for treating GIT disorders and as drug-delivery vehicles (33). Because of increase in antibiotic resistance, probiotics and their products, such as bacteriocins, promise to be good alternatives as antimicrobials. Globally, many communities recognize the importance of probiotic microorganisms as evidenced by increasing demands for dairy products, especially yoghurt. Due to increasing food prices, communities in rural and urban areas in developing countries should be encouraged to continue consuming their household fermented foods and beverages as part of normal diet to exploit the probiotic benefits of these products.

Food products that simultaneously contain probiotics and prebiotics are called synbiotics. This combination ensures the survival of probiotics through the gut and facilitates their delivery into the large intestine. Prebiotics stimulate the growth and activity of endogenic intestinal microflora (33). Most African traditional fermented foods and beverages fit the synbiotics definition perfectly, as they comprise of residual stomach-indigestible polysaccharides together with the LAB used for fermentation. Recent advancements in the science of probiotics research at a molecular level include using high throughput sequencing techniques. This will enable researchers to decipher unique probiotic gene products with novel applications. The use of probiotics offers an innovative approach for developing formulations applied as functional foods for managing diseases such as chronic inflammatory gastrointestinal disorders and many lifestyle diseases.

## Concluding remarks

The value of fermented food cannot be overemphasized, as they have always played a vital role in diet and nutrition. The benefits associated with fermented foods, such as increased shelf life, palatability, and nutritional value, suggest the importance of these foods. The probiotic effects of various fermented foods and their associated microbial cultures look promising and warrant further research. Fermentation products of probiotic LAB from traditional foods can be a suitable alternative source of antimicrobial agents, incorporated in the fight against emerging antibiotic-resistant microorganisms. Food fermentation is a method of preserving food by presenting a hurdle against pathogenic bacteria, and can mitigate diarrhea in communities with inferior potable water quality. Traditional foods, appropriately produced, are safe and acceptable, and qualify as a source of probiotics with novel applications.

While the mechanisms of action of probiotic LAB and their products, including bacteriocins, warrant further experimentation (*in vitro* and *in vivo*), and controlled clinical trials to exploit the maximum value is important, current developments suggest that consumption of fermented foods and beverages should be encouraged in all communities, especially in developing countries where probiotic dairy products may come at a great price. Probiotic bacteria have a potential in solving current and emerging lifestyle diseases. Surprisingly, LAB from fermented foods were recently found to play a positive role in mental health. Most of the probiotic microbial cultures in literature and currently available in the market are from outside Africa even though this continent has such a rich diversity of fermented foods that needs to be exploited as a source of probiotics. Hence, the search for novel probiotic bacteria from African fermented foods and beverages; and from other parts of the world should be intensified. Finally, metagenomics can play a crucial role in this process by providing insights into the genes, structure, and function of products from probiotic microorganisms.
